# Flucytosine Pharmacokinetics in a Critically Ill Patient Receiving Continuous Renal Replacement Therapy

**DOI:** 10.1155/2015/927496

**Published:** 2015-07-12

**Authors:** Megan E. Kunka, Elizabeth A. Cady, Heejung C. Woo, Melissa L. Thompson Bastin

**Affiliations:** ^1^University of Kentucky HealthCare, 800 Rose Street, H110, Lexington, KY 40536, USA; ^2^University of Kentucky, Lexington, KY 40506, USA

## Abstract

*Purpose*. A case report evaluating flucytosine dosing in a critically ill patient receiving continuous renal replacement therapy. *Summary*. This case report outlines an 81-year-old male who was receiving continuous venovenous hemofiltration (CVVH) for acute renal failure and was being treated with flucytosine for the treatment of disseminated *Cryptococcus neoformans* infection. Due to patient specific factors, flucytosine was empirically dose adjusted approximately 50% lower than intermittent hemodialysis (iHD) recommendations and approximately 33% lower than CRRT recommendations. Peak and trough levels were obtained, which were supratherapeutic, and pharmacokinetic parameters were calculated. The patient experienced thrombocytopenia, likely due to elevated flucytosine levels, and flucytosine was ultimately discontinued. *Conclusion*. Despite conservative flucytosine dosing for a patient receiving CVVH, peak and trough serum flucytosine levels were supratherapeutic (120 *μ*g/mL at 2 hours and 81 *μ*g/mL at 11.5 hours), which increased drug-related adverse effects. The results indicate that this conservative dosing regimen utilizing the patient's actual body weight was too aggressive. This case report provides insight into flucytosine dosing in CVVH, a topic that has not been investigated previously. Further pharmacokinetic studies of flucytosine dosing in critically ill patients receiving CVVH are needed in order to optimize pharmacokinetic and pharmacodynamic parameters while avoiding toxic flucytosine exposure.

## 1. Background

Amphotericin and flucytosine are the first-line agents for the treatment of cryptococcal meningoencephalitis [1]. Dosing of flucytosine is weight based and is typically 25 mg/kg/dose every 6 hours for at least 4 weeks for cryptococcal meningoencephalitis in non-HIV infected, nontransplant patients. A shorter, two-week course of induction therapy may be considered for patients who have low risk of therapeutic failure, who were previously healthy with no underlying, uncontrolled disease states, and who have had an excellent clinical response to the initial two-week combination. Six weeks of induction therapy may be required in patients with neurological complications. Consolidation and then maintenance therapy are recommended following the induction stage, for a total of 6–12 months of treatment. The Infectious Diseases Society of America (IDSA) Guidelines elaborate on numerous caveats in the event that patients are unable to tolerate the first-line antifungal combination for cryptococcal infections.

Regarding flucytosine dosing in patients with severe renal impairment requiring renal replacement therapy, limited evidence exists regarding the appropriate dosing. In fact, a boxed warning, a caution issued by the U.S. Food and Drug Administration (FDA) regarding serious adverse drug reactions, suggests extreme caution be utilized if flucytosine is warranted in patients with renal dysfunction [2].

Flucytosine (5-fluorocytosine; 5-FC) is one of the oldest antifungal agents on the market. Initially synthesized in 1957 as a potential antitumor agent, flucytosine was later approved to treat human candidiasis and cryptococcosis [3]. Flucytosine penetrates susceptible fungal cells where it is converted to fluorouracil (5-FU) via the enzyme cytosine deaminase [2–4]. Fluorouracil is converted into several active metabolites that falsely incorporate into the fungal RNA or interfere with fungal DNA, ultimately inhibiting fungal protein synthesis [2]. Flucytosine is readily absorbed with a bioavailability of 75–90% following oral administration [5, 6]. In the United States, flucytosine is only available in oral capsule dosage form (250 mg, 500 mg capsules) [2]. Due to the high water solubility, flucytosine is not well distributed into adipose tissues, with a volume of distribution range of 0.6–0.9 L/kg at steady state [6–9]. Due to the limited distribution, dosing in obese patients should be based upon ideal body weight [4]. Distribution sites of flucytosine include the cerebrospinal fluid (CSF), aqueous humor, joints, and peritoneal fluid. In fact, CSF concentrations can reach up to 60–90% of serum flucytosine concentrations [5]. Flucytosine is minimally protein bound, approximately 2–4%, and it undergoes minimal hepatic metabolism with more than 90% of the drug excreted unchanged in the urine, resulting in the need for renal dosage adjustments [4, 5]. The elimination half-life of flucytosine is typically 2–5 hours in patients with normal renal function; however, the half-life can increase up to 250 hours in patients with end stage renal disease or patients who are anuric [9]. No specific renal dose adjustment is recommended per the package insert directly, but the manufacturer recommends dose reduction in patients with impaired renal function. In patients receiving intermittent hemodialysis (iHD), it is recommended to administer flucytosine after dialysis, as flucytosine is dialyzable [8, 9]. Data for dosing flucytosine in continuous renal replacement therapy are scarce. See Table 1 for flucytosine dosing recommendations, which are dependent upon flucytosine total daily dose of either 100 mg/kg or 150 mg/kg [8, 9]. Utilizing recommended dose adjustments is pertinent, as flucytosine is associated with potentially severe adverse effects. Hepatotoxicity and bone marrow suppression (agranulocytosis, aplastic anemia, leukopenia, pancytopenia, and thrombocytopenia) are life-threatening conditions that can occur as a result of flucytosine therapy, which limits use in certain patients. Additional adverse effects of flucytosine include cardiac toxicity, central nervous system toxicity, and renal abnormalities.

*In vivo* studies indicate time above minimum inhibitory concentration (MIC) is the pharmacodynamic parameter most correlated to outcome with flucytosine monotherapy [3, 4]. Peak flucytosine efficacy has been reported at serum concentrations four times the MIC [6, 7]. The standard flucytosine dose of 100–150 mg/kg/day in non-HIV infected patients targets serum concentrations of ten times the MIC breakpoint of 4 *μ*g/mL. This suggests that much higher serum flucytosine concentrations are being obtained than required for optimal efficacy.

Current data suggest obtaining a peak flucytosine level once at steady state (after 3–5 doses), 2 hours after the oral dose [6]. Based on available literature, the peak flucytosine level should be 30–100 *μ*g/mL for the treatment of cryptococcal infections, and target flucytosine trough levels should be 25–50 *μ*g/mL [3, 4]. Flucytosine concentrations above 100 *μ*g/mL should be avoided, as levels that elevated have been associated with a greater incidence of thrombocytopenia and elevated liver enzymes [3, 4]. A general recommendation is to maintain flucytosine levels above 25 *μ*g/mL, but under 100 *μ*g/mL, in order to optimize pharmacodynamic parameters and avoid toxicity, although other sources suggest steady-state serum flucytosine goal ranges of 50–100 *μ*g/mL [4, 5].

The mode of renal replacement therapy plays a major role in the amount and type of medications that are removed during the dialysis session.

Key factors that determine the amount of drug removed by CVVH are surface area of the filter, type of filter, blood flow rate, replacement fluid rate, and location of replacement fluid entry, prefilter or postfilter [10]. Drugs that are readily cleared by CVVH are of low molecular weight, <500 Daltons, and low protein binding, <80% protein bound, with a small volume of distribution (Vd) ≤0.7 L/kg [11]. Flucytosine's pharmacokinetic properties mostly match that of drugs readily cleared by dialysis, as it is 129.1 Daltons in molecular weight and only 3-4% protein bound; however, the Vd has been reported as up to 0.9 L/kg in some studies (0.6–0.9 L/kg), which may limit the amount of drug available to be removed by convection.

## 2. Case Report

An 81-year-old Caucasian male presented to the emergency department with shortness of breath and hemoptysis. Over the previous one to two months, the patient experienced fatigue and increasing shortness of breath. Episodes of hemoptysis were two to four times per day. His past medical history included ureteral and bladder cancer, recurrence of papillary tumor and prostatic urethra status after right nephroureterectomy and chemotherapy, deep vein thrombosis, hypertension, type 2 diabetes mellitus, and hypothyroidism. Regarding social history, the patient farmed full-time in his retirement, and he had recently cleaned out his chicken coop, which contained several months of built-up feces and straw bedding.

Upon admission, initial laboratory values included sodium 143 mEq/L, potassium 4.2 mEq/L, chloride 106 mEq/L, blood urea nitrogen 58 mg/L, creatinine 4.29 mg/dL, glucose 118 mg/dL, white blood cell count 9.7 × 10^3^/mm^3^, hemoglobin 6.4 g/dL, and platelet count 169 × 10^3^/mm^3^. Liver function test values were alanine aminotransferase 6 U/L, aspartate aminotransferase 13 U/L, and alkaline phosphatase 55 U/L. The patient was on warfarin for deep vein thrombosis, and anticoagulation results indicated prothrombin time (PT) 108.2 seconds, international normalized ratio (INR) 9.6, and activated partial thromboplastin time (aPTT) 49 seconds.

Numerous other laboratory tests were completed, including cytoplasmic anti-neutrophil cytoplasmic antibodies (c-ANCA), perinuclear anti-neutrophil cytoplasmic antibodies (p-ANCA), glomerular basement membrane antibody IgG, urine* Histoplasma*/*Legionella*/*Streptococcus pneumoniae* antigens, QuantiFERON tuberculosis gold, and beta-glucan. All of these tests were negative. Of note, beta-glucan test does not reliably detect* Cryptococcus*, Zygomycetes, or* Blastomyces dermatitidis*. Abnormal laboratory values included c-reactive protein 8.9 mg/dL, automated sedimentation rate >120 mm/hr, N-terminal pro-brain natriuretic peptide (NT-proBNP) 9,789 pg/mL, and troponin T 0.106 ng/mL.

The initial differential diagnosis and assessment included inflammatory pneumonitis, infection, and immunologic pathology. The patient was initiated on ceftriaxone and azithromycin, and a respiratory culture was obtained. Throughout the initial hospital course, his oxygen saturation dropped on a nonrebreather mask, and the patient was agreeable to elective intubation with bronchoscopy. Antibiotics were broadened to levofloxacin, piperacillin/tazobactam, and vancomycin, and corticosteroids were added. Cystogram was completed per urology which showed reflux with nonobstructive hydronephrosis, and nephrology was consulted to rule out other causes of the patient's acute kidney injury, none of which were definitive diagnoses. On hospital day 19, the patient's blood cultures were positive for yeast, which was presumed to be* Cryptococcus neoformans*, and liposomal amphotericin B 300 mg (3.1 mg/kg actual body weight) daily was initiated. On hospital day 23,* Cryptococcus neoformans* was identified in the blood via matrix assisted laser desorption/ionization-time-of-flight (MALDI-TOF) mass spectrometry and confirmed via a biochemical method (API 20C strip). A lumbar puncture was completed which also grew* Cryptococcus neoformans*. Flucytosine was initiated on day five of liposomal amphotericin B treatment (hospital day 23).

As the patient was receiving intermittent hemodialysis at the time of flucytosine initiation, a dose of 2,500 mg (25.8 mg/kg actual body weight) every Tuesday, Thursday, and Saturday after dialysis sessions was chosen. See Table 2 for a schedule of the hemodialysis sessions the patient received. On hospital day 27, the patient was initiated on continuous renal replacement therapy due to hemodynamic instability with the addition of multiple vasopressors (norepinephrine, phenylephrine, and vasopressin). The patient remained intubated, sedated, and paralyzed for facilitation of mechanical ventilation. Continuous venovenous hemofiltration (CVVH) was initiated (with citrate for anticoagulation) as the primary mode of dialysis and was maintained on the following settings: total replacement fluid rate, 2,500 mL/hr; blood flow rate, 300 mL/min; preblood pump rate (PBP) (citrate flow rate), 450 mL/hr; and ultrafiltration rate, 100–400 mL/hr. Our institution utilizes the Gambro PrismaSATE system with the PrismaSATE HF 1400 polyarylethersulfone filter. Due to inability to ultrafiltrate with CRRT at all times, the average fluid removal rate was 265 mL/day (11 mL/hr) over 3 days (hospital days 27–29).

As a result of the dialysis change to CVVH, the flucytosine dose was increased to 2,500 mg (25.8 mg/kg actual body weight) by mouth every 12 hours. Of note, this flucytosine dose was significantly conservative, as it utilized a total daily dose of 50 mg/kg/day, as compared to some literature that recommends up to 150 mg/kg/day divided. The choice for conservative flucytosine dosing was made based on the patient's baseline thrombocytopenia; the patient's platelet count was 39 × 10^3^/mm^3^ the day CVVH was initiated. Over the 3 days of CVVH, the patient had a mean urine output of approximately 0.21 mL/kg/day, classified as nonoliguric renal failure.

Flucytosine was discontinued on hospital day 29 due to thrombocytopenia, and high dose fluconazole was added. Liposomal amphotericin was replaced with conventional amphotericin on hospital day 37. The patient had negative blood cultures on hospital days 32 and 34.

The patient passed away on hospital day 47 from refractory hypoxemic respiratory failure, as life prolonging efforts were withdrawn per family request.

## 3. Methods

As the patient was overweight and not quite obese (body mass index, 29.7 kg/m^2^), flucytosine dosing was based upon the patient's actual body weight of 97 kg. The patient was initiated on flucytosine 2,500 mg by mouth every 12 hours on hospital day 27. An initial flucytosine level was ordered without regard to scheduled flucytosine doses (2 hours prior to the dose while the patient was receiving CVVH), and it resulted at 90 *μ*g/mL (hospital day 28). Peak and trough flucytosine levels were obtained after the fifth dose of the CVVH flucytosine regimen. Of note, prior to that, the patient had received three doses of flucytosine 2,500 mg after intermittent hemodialysis sessions. The peak level was obtained two hours after the fifth dose of flucytosine, and the trough level was obtained 30 minutes prior to the next scheduled dose of flucytosine (11.5 hours after the previously administered dose). These peak and trough levels were obtained on hospital day 29, but the results were not reported until hospital day 38. Due to the thrombocytopenic toxicity of flucytosine experienced by the patient, flucytosine was discontinued on hospital day 29, prior to the results of the peak and trough levels.

## 4. Results

The patient's flucytosine peak level resulted at 120 *μ*g/mL, and the flucytosine trough level was 81 *μ*g/mL. Pharmacokinetic calculations indicate an elimination rate constant (ke) of 0.04 hr^−1^, with a half-life (*t*1/2) of 16.75 hours [12]. The volume of distribution was 48.1–57.7 L (0.50–0.59 L/kg, range based on bioavailability of 75–90%), the area under the curve (AUC) was 2,980 *μ*g·hr/mL, and the total clearance was 1,924–2,308 mL/hr (range based on bioavailability of 75–90%). The minimum inhibitory concentration (MIC) for flucytosine was reported as 2 *μ*g/mL; however, it was noted that no Clinical Laboratory Standards Institute (CLSI) range exists for flucytosine and* Cryptococcus neoformans* for susceptibility interpretation. Results of the MIC are for research use only. The patient's serum flucytosine concentrations were well above the concentration associated with peak flucytosine efficacy, four times the MIC or 8 *μ*g/mL.

**Figure 1:**
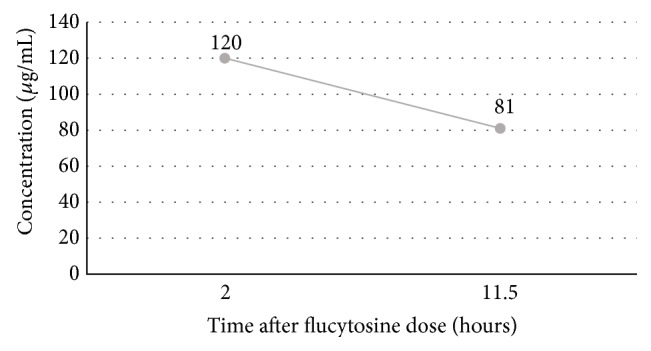
Flucytosine concentration versus time.

Figure 1 depicts the patient's flucytosine concentrations.

**Figure 2:**
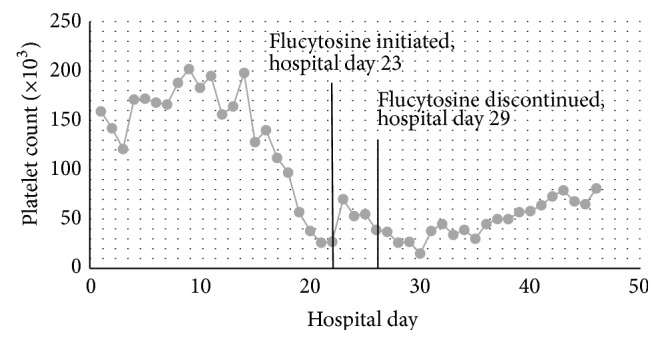
Platelet count trend.

As a result of the patient's declining platelet count, flucytosine was discontinued on hospital day 29 (platelet count of 26 × 10^3^/mm^3^), 48 hours after CVVH was initiated.  Figure 2 depicts the platelet count trend for the patient. The nadir platelet count was 15 × 10^3^/mm^3^, which occurred on hospital day 31, 9 days after initiation of flucytosine. The patient was receiving concomitant medications that could contribute to thrombocytopenia during this time, including amphotericin, cefepime, famotidine, metronidazole, and vancomycin.

Despite the elevated flucytosine levels, the patient did not experience any hepatotoxicity. In fact, the patient's liver function tests throughout the course of his stay were lower than or within normal range.

## 5. Discussion

The patient described above had a supratherapeutic flucytosine peak concentration of >100 *μ*g/mL and higher than expected levels overall. Potential reasons for the higher flucytosine levels include potential accumulation of flucytosine during the hemodialysis period, limited fluid removal from CVVH, and limited time receiving CVVH before flucytosine discontinuation (<72 hours). The initial flucytosine level of 90 *μ*g/mL, albeit drawn 2 hours prior to the flucytosine dose, indicates that the patient was likely accumulating flucytosine while receiving intermittent hemodialysis, as this level was obtained after only 4 flucytosine doses (2 iHD doses and 2 CRRT doses). As the method of CVVH utilizes a convection-only method of solute removal, this may have limited the flucytosine removal and resulted in higher than expected flucytosine levels. Flucytosine has a small molecular weight (129.1 Daltons), which could explain the lower flucytosine clearance seen with convection, as low molecular weight solute is more efficiently removed by diffusion modalities such as continuous venovenous hemodialysis (CVVHD) or iHD.

Another potential reason for the flucytosine toxicity is the use of the patient's actual body weight for flucytosine dosing. Although the patient was not technically obese, the patient was overweight, and potentially utilizing adjusted (80 kg) or ideal body weight (75 kg) would have resulted in therapeutic flucytosine levels. Dodds and colleagues suggest utilizing ideal body weight in obese patients; however, limited literature exists regarding the weight utilized for flucytosine dosing in patients who are overweight, and consensus does not exist.

The patient was receiving multiple vasopressors as well as trophic tube feeds during the time of flucytosine administration and CVVH. Food decreases the rate of flucytosine absorption, but it does not impact the extent of absorption [5]. Therefore, we believe the trophic tube feeds had minimal impact on the patient's flucytosine concentrations.

In comparing the patient-specific flucytosine pharmacokinetics to what is documented in the literature, the patient had a lower volume of distribution (0.50–0.59 L/kg) than the general population (0.6–0.9 L/kg). Our observed reduced volume of distribution offers no explanation as to the reduced flucytosine clearance.

As this is the first published case of its kind regarding flucytosine dosing in CVVH, we believe our findings make a significant contribution to the critical care literature.

## 6. Conclusion

Flucytosine is recommended as a component of the first-line antifungal combination for the treatment of cryptococcal infections. Despite its use for many years, optimizing flucytosine dosing in critically ill patients on renal replacement therapy remains difficult, as clinical data are lacking in this patient population. In our patient summarized above, the total clearance of flucytosine by CVVH was less than expected and resulted in significant hematologic toxicities, warranting drug discontinuation. Our patient experienced significant toxicities related to the supratherapeutic flucytosine levels, even though the empiric dosing regimen was conservative, approximately 1/2 the recommended iHD dose and 1/3 the recommended CRRT dose [3, 5, 8–10]. As the patient was overweight, yet not obese, potentially utilizing a lower dosing regimen with the patient's adjusted or ideal body weight would have resulted in therapeutic serum levels and reduced the risk of toxicity. As flucytosine is a mainstay of treatment for patients with severe cryptococcal disease, it is paramount that dosing recommendations more accurately reflect the real world experience with CRRT dosing. This case report provides insight into flucytosine dosing in CVVH, a topic that has not been investigated previously in the literature. Additional pharmacokinetic studies are needed to further explore the pharmacokinetics of flucytosine removal with CVVH, as the dosing regimens currently recommended may be supratherapeutic and result in unwanted toxicities.

## Figures and Tables

**Table 1:** Flucytosine dosing recommendations in renal impairment^a^  [3, 5, 8–10].

Flucytosine dosing of 100 mg/kg/day (normal renal function)		Flucytosine dosing of 150 mg/kg/day (normal renal function)	
CrCL (mL/min)	Dose	CrCL (mL/min)	Dose
>50	25 mg/kg every 6 hours	>40, >50	37.5 mg/kg every 6 hours
10–50	25 mg/kg every 12–24 hours	20–40, 10–50	37.5 mg/kg every 12–24 hours
<10	25 mg/kg every 24–48 hours	<20, <10	37.5 mg/kg every 24–48 hours
Hemodialysis	Single, supplemental doses of 20–50 mg/kg after dialysis sessions	Hemodialysis	Single, supplemental doses after dialysis sessions
CRRT	No specific recommendations	CRRT	CVVHD/CVVH: 37.5 mg/kg every 12–24 hours

^a^CrCL: creatinine clearance, CRRT: continuous renal replacement therapy, and CVVHD/CVVH: continuous venovenous hemodialysis/continuous venovenous hemofiltration.

**Table 2:** Hemodialysis sessions and corresponding flucytosine administrations.

Hospital day	Event
14	Intermittent hemodialysis session
18	Intermittent hemodialysis session
20	Partial intermittent hemodialysis session
22	Intermittent hemodialysis session
23	Flucytosine 2,500 mg given
25	Intermittent hemodialysis session Flucytosine 2,500 mg given
